# Impulse control disorders in patients with pituitary adenoma managed with or without dopamine agonists: a cross-sectional study from a UK centre

**DOI:** 10.1007/s11102-025-01517-0

**Published:** 2025-04-21

**Authors:** Ross Hamblin, Mary White, Athanasios Fountas, Niki Karavitaki

**Affiliations:** 1 Department of Metabolism and Systems Science, College of Medicine and Health, University of Birmingham, IBR Tower, Level 2, Birmingham, B15 2TT UK; 2 Centre for Endocrinology, Diabetes and Metabolism, Birmingham Health Partners, Birmingham, UK; 3 Department of Endocrinology, Queen Elizabeth Hospital, University Hospitals Birmingham NHS Foundation Trust, Birmingham, UK

**Keywords:** Pituitary adenoma, Dopamine agonist, Impulse control, Prolactinoma, Acromegaly

## Abstract

**Purpose:**

Studies from various countries raise concerns on the association between dopamine agonist (DA) treatment and impulse control disorders (ICDs) in patients with pituitary adenomas. We investigated the prevalence of ICDs in patients with pituitary adenomas in a UK centre using two instruments applied in clinical practice for assessing ICDs.

**Methods:**

Cross-sectional study of adults with prolactinoma, acromegaly or non-functioning pituitary adenoma (NFPA) treated or not with DAs in a pituitary centre. Screening tools for ICD were the Minnesota Impulse Disorders Interview (MIDI) and Questionnaire for Impulsive-Compulsive Disorders in Parkinson’s Disease Rating Scale (QUIP-RS).

**Results:**

Data from 200 patients were analysed [72 prolactinomas (on DA), 71 NFPAs (no DA), 57 with acromegaly (12 on DA)]. The percentage of patients scoring for any ICD was higher in the prolactinoma on DA group compared with the NFPA cases; MIDI 12.7% vs. 1.4% (*p* = 0.02) and QUIP-RS 46.5% vs. 18.6% (*p* < 0.001), respectively. DA use was associated with positive scores for all ICDs in the MIDI and with compulsive buying and hobbyism-punding in the QUIP-RS. DA dose or treatment duration were not associated with positive scores. The responses of patients with acromegaly on DA did not differ from those not on these agents in any questionnaire.

**Conclusions:**

In this sample of UK patients, prevalence of ICDs is higher in prolactinoma patients on DA compared to those with NFPA not on DA. Differences were not observed between patients with acromegaly treated or not with DA. Different ICD assessment tools have an impact on the detected outcomes.

## Introduction

Impulse control disorders (ICDs) comprise a group of behaviours defined by an inability to resist an impulse, drive or temptation to perform an act considered to be harmful to an individual or others [1, 2]. ICDs include (but are not exclusive to) hypersexuality, compulsive eating, gambling disorder/pathological gambling and compulsive buying, whilst related behaviours include hobbyism, punding (repetitive and purposeless behaviours) and dopamine dysregulation syndrome (compulsive medication use) [3]. Increasingly, these disorders have been reported in patients with pituitary adenomas treated with dopamine agonists (DAs) [4–18]. DAs are commonly offered in the management of pituitary adenomas, primarily for patients with prolactinoma, but also in selected patients with acromegaly, non-functioning pituitary adenoma (NFPAs) and Cushing’s disease [19–21]. Numerous reports, mainly concerning patients with Parkinson’s disease (PD) but also those with pituitary adenoma on DAs, have highlighted the considerable personal, social and financial impact of ICDs on them and their families [14, 22–26]. Further understanding of the association between DA use and ICDs in patients with pituitary adenoma is needed to adequately clarify the potential adverse sequelae of this treatment approach; this is particularly important, given that pituitary surgery has become an increasingly favourable alternative for selected patients with prolactinoma [27].

Most evidence associating DAs with ICDs originates from studies of patients with PD [28–30]. Indeed, in this group, a higher prevalence of ICDs has been demonstrated in those on DA, both when compared to patients not treated with DA or to the background population [28, 31]. Comparatively, evidence underpinning an association between DAs with ICDs specifically in patients with pituitary adenomas is often inconclusive. In a recent systematic review, the prevalence of ICDs ranged between 0% and 60% in cases of prolactinoma and between 5% and 23% for those with acromegaly [15]. Uncertainty and reported variation in the ICD prevalence are driven by limitations within studies, most of which have been prone to selection bias [4, 8, 32], challenged by small sample sizes or few case events [7, 11–14], or lacked control groups of patients with pituitary adenoma [8, 9, 14–16, 32, 33]. Inclusion of appropriate controls, i.e., patients with pituitary adenoma without exposure to DA, is needed to establish whether ICDs observed are related with DA treatment alone or the pituitary adenoma, *per se.* Such distinction is pertinent given that different pituitary adenoma subtypes have been associated with variable degrees of impulsiveness, and patients with pituitary adenoma have been shown to be more likely to experience mental health issues compared to the background population [34–36].

To address these limitations, we conducted a large cross-sectional study investigating the prevalence of ICDs in patients with prolactinoma or acromegaly or non-functioning pituitary adenoma (NFPA) treated with or without DA by using two instruments applied in clinical practice for assessing ICDs.

## Materials and methods

This cross-sectional study was conducted in the Department of Endocrinology, Queen Elizabeth Hospital, University Hospitals Birmingham NHS Foundation Trust, Birmingham, UK. Eligible patients included subjects over the age of 18 with a diagnosis of prolactinoma or acromegaly or non-functioning pituitary adenoma, treated with or without DA. Exclusion criteria were known history of ICD, use of DA for a condition other than pituitary adenoma, and inability to complete the questionnaire or to provide informed consent. Participants with a current history of a mental health disorder requiring medical treatment were also excluded. Eligible participants were approached and informed about the study during routine clinical outpatient appointments. Participants who subsequently agreed to participate completed two questionnaires used to explore for ICDs in clinical practice. These included a modified version of the Minnesota Impulse Disorders Interview (MIDI), a semi-structured interview tool with questions assessing hypersexuality, gambling disorder and compulsive buying [37, 38]. Scoring was calculated in line with recommendations by Grant et al. (2005) [38]; a positive screen for compulsive buying was defined as affirmative responses to each question within the module, a positive screen for gambling disorder was represented by at least five affirmative responses, and a positive screen for hypersexuality was given if the subject provided affirmative responses to one of two questions. The second ICD screening tool, the Questionnaire for Impulsive-Compulsive Disorders in Parkinson’s Disease–Rating Scale (QUIP-RS) [39], is applied to screen for various ICDs and related behaviours. This brief assessment uses a 5-point Likert scale (never to very often) to assess the frequency of participant engagement in behaviours linked to ICDs. The following threshold scores, established by the instrument authors were applied: a score of ≥ 6 was regarded as a positive screen for pathological gambling, ≥ 8 for compulsive buying, ≥ 8 for hypersexuality, ≥ 7 for compulsive eating, and ≥ 7 for hobbyism-punding [39]. In addition, those patients who met the threshold scores for any individual ICD category were classified as “positive for any ICD”.

Demographic and clinical data were collected from questionnaires and medical records. Secondary hypogonadism was defined as low or inappropriately normal levels of follicle-stimulating hormone (FSH) and luteinising hormone (LH) coupled with low testosterone in males or low oestradiol and oligo/amenorrhoea in pre-menopausal females or as gonadotropins below the reference range in post-menopausal women; secondary hypothyroidism was defined as a free thyroxine (FT4) level below the reference range combined with a low, or inappropriately normal thyroid-stimulating hormone (TSH) level. Secondary hypoadrenalism was confirmed by dynamic testing (short Synacthen or insulin tolerance test). In patients with acromegaly, the disease was considered active if the IGF-1 was above the reference range.

The study was granted ethical approval by the London Central Research Ethics Committee (Reference 19/LO/0478) and was conducted between October 2019 and September 2022 but was paused between March 2020 and February 2021 due to the COVID-19 pandemic. Following the changes in consultation practice adopted during that period, the study protocol was amended (after ethical approval) to allow remote recruitment via telephone consultation.

### Statistical analyses

Percentages were used for categorical variables and median with ranges for continuous variables. The Chi-square test was applied to compare categorical variables between the groups of patients; if greater than 20% of expected values were less than 5, Fisher’s exact test was used instead. Continuous variables were compared using the Mann-Whitney U-test. Regression analysis was used to investigate the association between various parameters and the presence of ICDs, and odds ratios (OR) with 95% confidence intervals (CI) were generated. Statistical significance was set at *p* < 0.05. Statistical analyses were generated using IBM SPSS Statistics for Mac, Version 29 (IBM Corp., Armonk, NY, USA).

## Results

Two hundred and fifty-four patients eligible for participation were approached; 14 declined to take part, and 32 who were initially receptive to the study did not return consent forms after written information about the study was provided. Due to limited number of cases, patients with prolactinoma not on DA (*n* = 6) and patients with NFPA treated with DA (*n* = 1) were removed from further analyses. Of the 201 enrolled subjects, one patient with a prolactinoma on DA was subsequently excluded as neither ICD assessment tools (MIDI or QUIP-RS) were correctly completed. Two hundred patients (72 with prolactinoma, 71 with NFPA and 57 with acromegaly) were suitable for analysis.

### Patient characteristics

#### Patients with prolactinoma or NFPA

All 72 patients with prolactinoma were on DA during the participation in the study, 97% of whom were taking cabergoline at a median dose of 0.5 mg/week (range 0.25-4 mg) over a median duration of 103 months (range 1-498); none of the 71 patients with NFPA were on DA. Characteristics of the two groups are shown in Table 1. At the time of assessment, all males and pre-menopausal females with FSH/LH deficiency were on relevant hormonal replacement, except for two males with prolactinoma.

**Table 1:** Characteristics of patients with prolactinoma or NFPA

	Prolactinoma	NFPA	*p* value
Number of patients	72	71	
Number of patients on DA	72	0	
DA type during assessment, n (%)			
Cabergoline	70 (97.2%)	N/A	
Bromocriptine	2 (2.8%)	N/A	
Duration of treatment with cabergoline, months, median (range)	103 (1-498)	N/A	
Weekly dose of cabergoline at time of assessment, mg, median (range)	0.5 (0.25-4)	N/A	
Age, years, median (range)	50 (21–77)	60 (24–78)	< 0.001
Sex, males, n (%)	38 (52.8%)	37 (52.1%)	1.00
Treated with surgery, n (%)	5 (6.9%)	63 (88.7%)	< 0.001
Treated with radiotherapy, n (%)	0 (0%)	12 (16.9%)	< 0.001
Hypopituitarism, n (%)			
LH/FSH deficiency at time of assessment	15 (20.8%)	38 (53.5%)	< 0.001
LH/FSH deficiency at time of assessment (males only)	13 (34.21%)	22 (59.5%)	0.04
LH/FSH deficiency on replacement at time of assessment (males only)	11 (84.6%)	2 (100%)	0.26
TSH deficiency at time of assessment	8 (11.1%)	29 (40.8%)	< 0.001
TSH deficiency on replacement at time of assessment	8 (100%)	29 (100%)	
ACTH deficiency at time of assessment	6 (8.3%)	21 (29.6%)	0.001
ACTH deficiency on replacement at time of assessment	6 (100%)	21 (100%)	
In a relationship, n (%) *	45/69 (65.2%)	45/68 (66.2%)	1.00
Education, n (%) *			0.32
School +/- College only	42/67 (62.7%)	39/67 (58.2%)	
Professional qualification/diploma	11/67 (16.4%)	7/67 (10.4%)	
Higher education/University degree	14/67 (20.9%)	21/67 (31.3%)	
Working, n (%) *	48/70 (68.6%)	46/70 (65.7%)	0.86
Family history of mental health disorder, n (%) *	9/71 (12.7%)	4/69 (5.8%)	0.24
Ethnicity, n (%) *			0.22
Black British/Afro-Caribbean/Black other	11/72 (15.3%)	6/69 (8.7%)	
White British/White other	53/72 (73.6%)	57/69 (82.6%)	
Chinese	2/72 (2.8%)	0/69 (0%)	
South Asian	4/72 (5.6%)	6/69 (8.7%)	
Other	2/72 (2.8%)	0/69 (0%)	

*Denominators include the total number of patients who responded

NFPA = Non-functioning pituitary adenoma, DA = Dopamine agonist, LH = Luteinising hormone, FSH = Follicle-stimulating hormone, TSH = Thyroid-stimulating hormone, ACTH = Adrenocorticotrophic hormone

#### Patients with acromegaly

Fifty-seven patients had acromegaly, 12 of which were taking DA (all cabergoline). The median duration of DA treatment until assessment was 73 months (range 12–340 months), and the median weekly dose of DA at the time of evaluation was 1.25 mg (range 0.25-3 mg). Characteristics of the two groups are shown in Table 2. At the time of assessment, all males and pre-menopausal females with FSH/LH deficiency were on relevant hormonal replacement except for one male and one pre-menopausal female (both not on DA). The number of patients with active acromegaly did not differ between those receiving DA and those not on this treatment (Table 2).

**Table 2:** Characteristics of the patients with acromegaly

	On DA	Not on DA	*p* value
Number	12	45	
DA type during assessment, n (%)			
Cabergoline only	11 (91.7%)	N/A	
Bromocriptine only	0 (0%)	N/A	
Cabergoline with prior treatment with bromocriptine	1 (8.3%)	N/A	
Duration of DA treatment, median, months (range)	73 (12–340)	N/A	
Weekly dose of cabergoline at time of assessment, mg, median (range)	1.25 (0.25-3)	N/A	
Age, years, median, (range)	58 (34–79)	58 (26–76)	0.35
Sex, males, n (%)	9 (75.0%)	15 (33.3%)	0.02
Treated with surgery, n (%)	7 (58.3%)	41 (91.1%)	0.02
Treated with radiotherapy, n (%)	5 (41.7%)	13 (28.9%)	0.49
Hypopituitarism, n (%)			
LH/FSH deficiency at time of assessment	5 (41.7%)	20 (44.4%)	1.00
LH/FSH deficiency at time of assessment (males only)	5 (55.6%)	5 (33.3%)	0.40
LH/FSH deficiency on replacement at time of assessment (males only)	5 (100%)	4 (80.0%)	1.00
TSH deficiency at time of assessment	5 (41.7%)	12 (26.7%)	0.48
TSH deficiency on replacement at time of assessment	5 (100%)	12 (100%)	
ACTH deficiency at time of assessment	5 (41.7%)	14 (31.1%)	0.51
ACTH deficiency on replacement at time of assessment	5 (100%)	14 (100%)	
Active acromegaly at the time of assessment, n (%)	4 (33.3%)	9 (20.0%)	0.36
In a relationship, n (%) *	6/12 (50.0%)	31/44 (70.5%)	0.30
Education, n (%) *			0.49
School+/- college	9/11 (81.8%)	25/43 (58.1%)	
Professional qualification/diploma	1/11 (9.1%)	7/43 (16.3%)	
University degree	1/11 (9.1%)	11/43 (25.6%)	
Working, n (%)	7 (58.3%)	28 (62.2%)	1.00
Family history of mental health disorder, n (%) *	0/12 (0%)	11/43 (25.6%)	0.10
Ethnicity, n (%)			1.00
Black British/Afro-Caribbean/Black other	0 (0%)	3 (6.7%)	
White British/White other	12 (100%)	39 (86.7%)	
South Asian	0 (0%)	3 (6.7%)	

*Denominators include the total number of patients who responded.

DA = Dopamine agonist, LH = Luteinising hormone, FSH = Follicle-stimulating hormone, TSH = Thyroid-stimulating hormone, ACTH = Adrenocorticotrophic hormone

### Responses of patients with prolactinoma or NFPA

The percentages of patients with prolactinoma or NFPA scoring for any ICD or for subsections of the questionnaires are shown in Table 3.

**Table 3:** Prevalence of ICD in patients with prolactinoma, NFPA and acromegaly based on the MIDI and QUIP-RS questionnaires and comparison of scores between groups

	Any ICD MIDI	Any ICD QUIP-RS	Compulsive Buying MIDI	Compulsive Buying QUIP-RS	Pathological Gambling MIDI	Compulsive Gambling QUIP-RS	Hypersexuality MIDI	Hypersexuality QUIP-RS	Compulsive Eating QUIP-RS	Punding QUIP-RS
Patients with prolactinoma (all on DA), n (%) *	9/71 (12.7%)	33/71 (46.5%)	4/71 (5.6%)	11/71 (15.5%)	4/71 (5.6%)	6/70 (8.6%)	3/71 (4.2%)	9/71 (12.7%)	18/69 (26.1%)	24/70 (34.3%)
Patients with NFPA (none on DA), n (%) *	1/71 (1.4%)	13/70 (18.6%)	0/71 (0%)	1/69 (1.4%)	1/71 (1.4%)	1/70 (1.4%)	0/71 (0%)	4/70 (5.7%)	7/68 (10.3%)	7/70 (10.0%)
*p* value	0.02	< 0.001	0.12	0.004	0.37	0.12	0.25	0.24	0.02	< 0.001
Patients with acromegaly on DA, n (%) *	2/12 (16.7%)	5/11 (45.5%)	1/12 (8.3%)	3/10 (30.0%)	0/12 (0%)	0/11 (0%)	1/12 (8.3%)	1/11 (9.1%)	3/10 (30.0%)	5/11 (45.5%)
Patients with acromegaly not on DA, n (%) *	1/45 (2.2%)	15/45 (33.3%)	1/45 (2.2%)	4/45 (8.9%)	0/45 (0%)	0/45 (0%)	0/45 (0%)	2/45 (4.4%)	6/44 (13.6%)	11/45 (24.4%)
*p* value	0.11	0.50	0.38	0.10	-	-	0.21	0.49	0.34	0.26
Patients with acromegaly not on DA, n (%) *	1/45 (2.2%)	15/45 (33.3%)	1/45 (2.2%)	4/45 (8.9%)	0/45 (0%)	0/45 (0%)	0/45 (0%)	2/45 (4.4%)	6/44 (13.6%)	11/45 (24.4%)
Patients with prolactinoma (all on DA), n (%) *	9/71 (12.7%)	33/71 (46.5%)	4/71 (5.6%)	11/71 (15.5%)	4/71 (5.6%)	6/70 (8.6%)	3/71 (4.2%)	9/71 (12.7%)	18/69 (26.1%)	24/70 (34.3%)
*p* value	0.086	0.180	0.647	0.399	0.157	0.080	0.281	0.198	0.157	0.304

*Denominators include the total number of patients who responded.

ICD = Impulse control disorder, NFPA = Non-functioning pituitary adenoma, MIDI = Minnesota Impulse Disorder Interview, QUIP-RS = Questionnaire for impulsive compulsive disorders in Parkinson’s disease-rating scale, DA = Dopamine agonist

#### Comparison of responses of patients with prolactinoma vs. NFPA

##### MIDI

The percentage of patients scoring for any ICD was higher in those with prolactinoma on DA compared with the group of NFPA cases (12.7% vs. 1.4%, *p* = 0.02). When comparing the frequency of specific ICDs between these two groups, there were no differences for compulsive buying (5.6% vs. 0%), pathological gambling (5.6% vs. 1.4%), or hypersexuality (4.2% vs. 0%) (Table 3).

##### QUIP-RS

The percentage of patients scoring for any ICD, for compulsive buying, compulsive eating and hobbyism-punding was higher in the group of those with prolactinoma treated with DA compared with the group of NFPA cases (46.5% vs. 18.6%, *p* < 0.001, 15.5% vs. 1.4%, *p* = 0.004, 26.1% vs. 10.3%, *p* = 0.02 and 34.3% vs. 10.0%, *p* < 0.001, respectively). The percentage of pathological gambling and hypersexuality did not differ between the two groups (8.6% vs. 1.4%, and 12.7% vs. 5.7%, respectively) (Table 3).

#### Regression analysis

On univariate regression analysis, DA use was associated with a positive score for all ICDs in the MIDI questionnaire (OR 10.2, 95% CI 1.25–82.5), whereas age or sex were not (Table 4).

**Table 4:** Univariate logistic regression analysis of factors associated with positive scoring on the questionnaires

	Any ICD (MIDI)	Any ICD (QUIP-RS)	Compulsive Buying (QUIP-RS)	Compulsive Eating (QUIP-RS)	Hobbyism-Punding (QUIP-RS)
DA use	OR 10.16 (95% CI 1.25–82.5) *p* = 0.03	OR 3.81 (95% CI 1.77–8.16) *p* = < 0.001	OR 12.47 (95% CI 1.56–99.4) *p* = 0.02	OR 3.08 (95% CI 1.19–7.94) *p* = 0.02	OR 4.70 (95% CI 1.86–11.83) *p* = 0.001
Age	OR 0.96 (95% CI 0.918–1.002) *p =* 0.06	OR 0.97 (95% CI 0.946–0.995) *p* = 0.02	OR 0.950 (95% CI 0.910–0.989) *p* = 0.01	OR 0.96 (95% CI 0.926–0.985) *p* = 0.004	OR 0.98 (95% CI 0.952–1.005) *p* = 0.12
Sex	OR 2.26 (95% CI 0.561–9.133) *p* = 0.25	OR 1.12 (95% CI 0.55–2.26) *p* = 0.76	OR 0.61 (95% CI 0.18–2.03) *p* = 0.42	OR 0.68 (95% CI 0.29–1.63) *p* = 0.39	OR 1.15 (95% CI 0.52–2.56) *p* = 0.73

ICD = Impulse control disorder, MIDI = Minnesota Impulse Disorder Interview, QUIP-RS = Questionnaire for impulsive compulsive disorders in Parkinson’s disease-rating scale, DA = Dopamine agonist, OR = Odds ratio, CI = Confidence interval

On the QUIP-RS, DA use, and younger age were associated with scoring for at least one ICD; in contrast, sex was not. On multivariate analysis, only DA use remained a significant factor (OR 3.27, 95% CI 1.48–7.24). For the sub-category of compulsive buying, DA use and younger age were associated with a positive score, but on multivariate analysis, only DA use remained significant (OR 9.00 95% CI 1.09–74.6). For compulsive eating, DA use and younger age were associated with a positive score and on multivariate analysis, only younger age remained significant (OR 0.96, 95% CI 0.932–0.995). For hobbyism-punding, only DA use was associated with a positive score (OR 4.70, 95% CI 1.86–11.83) (Tables 4 and 5).

**Table 5:** Multivariate logistic regression analysis of factors associated with positive scoring on the questionnaires

	Any ICD (QUIP-RS)	Compulsive Buying (QUIP-RS)	Compulsive Eating (QUIP-RS)
DA use	OR = 3.27 (95% CI 1.48–7.24) *p* = 0.003	OR = 9.00 (95% CI 1.09–74.59) *p* = 0.04	OR = 2.21 (95% CI 0.809–6.01) *p* = 0.12
Age	OR = 0.981 (95% CI 0.956–1.008) *p* = 0.16	OR = 0.964 (95% CI 0.923–1.006) *p* = 0.09	OR = 0.963 (95% CI 0.932–0.995) *p* = 0.02

ICD = Impulse control disorder, QUIP-RS = Questionnaire for impulsive compulsive disorders in Parkinson’s disease-rating scale, DA = Dopamine agonist, OR = Odds ratio, CI = Confidence interval

Neither DA dose nor DA treatment duration were associated with a positive score on any of the ICD subsections on the MIDI or QUIP-RS (data not shown).

### Responses of patients with acromegaly

Based on the MIDI questionnaire, there were no differences in the percentages for any ICD between patients treated with DA and those managed without DA (16.7% vs. 2.2%). Similarly, there were no differences when comparing any of the MIDI ICD sub-sections (Table 3). Comparisons of responses between patients with acromegaly without exposure to DA and patients with prolactinoma on DA did not differ when assessed by the MIDI questionnaire (Table 3).

On the QUIP-RS questionnaire, there was no difference in the percentage for any ICD between patients with acromegaly treated with DA and those treated without DA (45.5% vs. 33.3%). There were also no differences when comparing any of the QUIP-RS subsections (Table 3). Comparisons of responses between patients with acromegaly without exposure to DA and patients with prolactinoma on DA did not differ when assessed by the QUIP-RS questionnaire (Table 3).

## Discussion

This is the first study exploring the prevalence of ICDs in a large cross-sectional sample of patients with pituitary adenoma living in the UK and is also the first to compare the prevalence of ICDs in patients with acromegaly based on their exposure to DA therapy. We found that the prevalence of ICDs – determined by two distinct assessment tools used in clinical practice – is higher in patients with prolactinoma treated with DA than those with NFPA who had not been on these agents. Significantly more patients with prolactinoma (all exposed to DA) scored for any ICD on both the MIDI and QUIP-RS and, based on the QUIP-RS alone, had a higher prevalence of compulsive buying, compulsive eating, and hobbyism-punding behaviours. By contrast, in patients with acromegaly, the prevalence of ICDs did not differ between those treated with and without DA.

Across all relevant studies published to date, ICDs have been described in 0–60% of patients with prolactinoma [15], whereas in reports using revised or modified versions of the MIDI specifically, this percentage is between 2% and 28% [7, 12–15, 33]. Comparatively, 12.7% of our patients with prolactinoma scored positive for any ICD based on assessment with the MIDI questionnaire. Most earlier reports using this tool had smaller sample sizes [7, 12–14] and were subject to biases, including the inclusion of a small number of male participants [12] or were conducted in countries with legal restrictions on gambling [7, 12, 13, 33], factors which may account for differences in prevalence observed. Notably, we observed a high prevalence of ICDs when using the QUIP-RS assessment tool (46.5%); in other studies using more than one ICD assessment method, differences in prevalence based on the assessment tool have similarly been found [7, 11–13]. The QUIP-RS is an ICD screening tool with similarities to its predecessor, the QUIP and its shortened version, the QUIP-S; these tools were used by De Sousa et al. [8] and Beccuti et al. [6] who, in Australian and Italian cross-sectional studies, respectively, reported a high prevalence of ICDs amongst patients with pituitary disease [60% [8] and 51% [6] of DA-treated patients (the majority having prolactinoma), respectively]. The QUIP-S and QUIP-RS screen for more ICDs than some other assessment methods, and as a highly sensitive screening tool, it can be associated with false positive responses [40], which may have contributed to the high prevalence observed. Irrespectively, the QUIP-RS is recommended by the International Parkinson and Movement Disorder Society for use for both ICD detection/screening and when assessing ICD severity over time [41].

Regression analysis in the total group of patients with prolactinoma or NFPA identified DA use as a variable associated with ICDs on both assessment tools. Earlier studies using a range of ICD screening methods have shown a significant association between ICDs and DA use in patients with pituitary adenomas; in the cross-sectional Italian study by Beccuti et al. [6], 132 patients with pituitary tumour or functional hyperprolactinaemia with current or previous exposure to DA demonstrated a higher frequency of ICD/ICD-related behaviour on the QUIP assessment tool, compared to a reference group of 58 patients with functional hyperprolactinaemia, pituitary tumours or hypothalamic disease without prior exposure to DA (51% vs. 31%, *p* < 0.01). In the cross-sectional US study by Bancos et al. [4], eligible patients with prolactinoma selected by postal survey were screened for ICDs using a four-part questionnaire comprised of multiple ICD assessment tools, and ICD prevalence was compared to a group of patients with NFPAs not on DAs. Hypersexuality was more common in patients with prolactinoma treated with DA (current or historical use) compared to NFPA controls; on subgroup analysis, men in the DA group were more likely to have an ICD compared to NFPA controls (27.7% vs. 3.7%, OR 9.94) [4]. In the Australian multi-centre cross-sectional study by De Sousa et al. [8] ICDs measured by the QUIP-S were more prevalent in hyperprolactinaemic patients (95% of them had prolactinoma) treated with DA, compared to healthy controls not on DA (61.1% vs. 42.4% *p* = 0.01). Finally, Mohammad et al. [16], using the QUIP ICD assessment tool, reported a higher prevalence of ICDs in southern Iraqi patients with prolactinoma treated with DAs compared to healthy controls (30% vs. 6.7%, *p* = 0.02). Whilst supportive of our results, the methodology of these studies deserves discussion; patients without pituitary adenoma were included in both DA-exposed and non-DA-exposed groups by Beccuti et al. [6]. Results from Beccuti et al. [6] and Bancos et al. [4] may be influenced by recall bias, as 18% and 19% of participants, respectively, were grouped as exposed to DA despite not being treated with these drugs at the time of ICD assessment. Moreover, the use of healthy controls by De Sousa et al. [8] and Mohammad et al. [16] limits clarity on whether the increased prevalence of ICDs observed relates with DA use or the underlying pituitary adenoma, per se. In this latter study [16], cultural and legal prohibitions, as the authors acknowledge, may have also resulted in the underreporting of some ICDs, including gambling and hypersexuality in females.

It is interesting to note that an association between DA use and ICD in patients with pituitary adenoma has not been confirmed in all reports [7, 10–13, 18, 32]. Reasons for the discrepancy between the findings of our study and these earlier observations may be attributed to differences in sample size and number of cases [7, 32]; in the cross-sectional prospective study from Turkey by Celik et al. [7], only 25 patients with prolactinoma on DA were included in the exposed group. The limited number of patients with pituitary adenoma within control groups [10–12, 32], the use of different screening tools to determine ICD prevalence [10, 11, 18, 32], and the varied geographical locations and populations studied (America [10], China [11], India [32], and Turkey [7, 12, 13, 18]) may also contribute to the differences in the reported results.

We did not observe differences in the prevalence of ICDs when comparing DA-exposed and non-DA-exposed patients with acromegaly. Interestingly, patients with acromegaly have been reported to have reduced impulsivity traits and novelty-seeking behaviour compared to both patients with NFPA and healthy controls [34]. Only two published studies to date have explored ICDs in patients with acromegaly; Ozkaya et al. [13] prospectively compared the prevalence of ICD in 40 patients with acromegaly and 40 with prolactinoma exposed to DA for at least six months to 38 patients with NFPAs and 32 healthy controls; 5% of those with acromegaly treated with DA were diagnosed with ICD (based on the MIDI-R and psychiatric assessment), whereas no ICDs were diagnosed in the controls [13]. Hinojosa-Amaya et al. [10], in a cross-sectional study of 13 patients with acromegaly, found that 23.1% of them had an ICD based on the Barratt Impulsiveness Scale (BIS-11) (score > 60). Neither study directly compared ICD prevalence in patients with acromegaly based on whether they were exposed to DA or not.

Interestingly, neither dose nor duration of DA therapy were associated with ICD prevalence, similarly observed in nearly all studies of patients with pituitary adenomas to date [15, 17]. Nonetheless, DA cessation (where possible) or dose reduction is recommended in the event of ICD, given the observed improvement in the severity of symptoms following this action [7, 9, 14, 33].

Strengths of our study include the large number of patients, the use of two assessment tools validated to detect ICDs in clinical practice, and the inclusion of patients with pituitary adenoma without exposure to DA in the control groups. A further strength is the direct comparison of ICDs in patients with acromegaly based on DA exposure. Limitations include potential non-response bias as some subjects who were approached chose not to enrol in the study. However, it is anticipated that this may have been ameliorated by our high response rate. Whilst we used assessment tools established and recommended for detecting ICDs in patients with Parkinson’s disease, no screening tool has been validated for specific use in patients with pituitary adenomas, and specialist psychiatrists did not formally review those with positive scores to confirm the diagnosis of ICD (a drawback also involving most other published studies on patients with prolactinoma [4, 6, 8–11]). By using NFPAs as a control group, we endeavoured to remove confounding effects from having a pituitary adenoma itself; it is unknown to what extent differences in pituitary adenoma subtype impacts the prevalence of ICD. Nonetheless, using patients with a prolactinoma not on DA as a control group would not be practically feasible. The number of patients with acromegaly treated with DA was small, and this may have posed challenges in detecting differences between the two groups. Whilst this is one of the most extensive studies of ICD prevalence in patients with pituitary adenomas, confidence intervals of ORs for some ICDs were wide.

In conclusion, in our cross-sectional study involving patients managed in a pituitary centre in the UK, we have shown that those with prolactinoma treated with DA have a higher prevalence of positive responses in questionnaires exploring ICD than patients with NFPA without exposure to DAs. DA treatment has been identified as a factor associated with positive scores in any ICD, both in the MIDI and the QUIP-RS questionnaires. Although statistical comparisons would not be feasible due to the different structure of the questionnaires, positive scores on QUIP-RS feature in higher percentages as opposed to those picked up by the MIDI, highlighting the impact of the different assessment tools on the outcomes and the results of studies on this topic. Within the constraints of the small sample size, in patients with acromegaly, percentages of positive responses in the questionnaires did not differ between DA-treated and non-treated subjects; larger studies will shed further light on this issue and clarify if the reduced impulsivity rates previously described in this group could also play a role. Clinicians should be familiar with less recognised ICDs/ICD-related behaviours (including punding – an ICD-related behaviour we observed to be significantly associated with DA use), given that these behaviours may be less noticeable compared to those such as compulsive shopping and compulsive gambling. Likewise, patients and family/significant others (where appropriate) should be educated and counselled sufficiently before starting DA therapy and during the course of this treatment.

## Data Availability

No datasets were generated or analysed during the current study.
